# Inhibition of Heat Shock-Induced H3K9ac Reduction Sensitizes Cancer Cells to Hyperthermia

**DOI:** 10.7150/ijbs.86384

**Published:** 2023-09-11

**Authors:** Huiyun Lin, Yihui Song, Lingjun Song, Zilong Geng, Runtan Cheng, Yinrui Lei, Fang Guo

**Affiliations:** 1 Key Laboratory of Systems Biomedicine, Shanghai Center for Systems Biomedicine, Shanghai Jiao Tong University, 800 Dong chuan Road, Shanghai 200240, China.; 2 Pathology Center, Shanghai General Hospital, Shanghai Jiao Tong University School of Medicine, 100 Hai-Ning Road, Shanghai 200080, China.

**Keywords:** heat shock, H3K9 acetylation, hyperthermia, cancer therapy

## Abstract

Heat stress, clinically known as hyperthermia, is a promising adjunctive modality in cancer treatment. However, the efficacy of hyperthermia as a monotherapy is limited and the underlying mechanism remains poorly understood. Targeting histone modifications is an emerging strategy for cancer therapy, but little is known regarding the role of heat stress in altering these modifications. Here, we report that heat shock inhibits H3K9 acetylation (H3K9ac) via histone deacetylase 6 (HDAC6) regulation. Heat shock inhibits the interaction between HDAC6 and heat shock protein 90 (HSP90), enhances nuclear localization of HDAC6, and promotes HDAC6 phosphorylation, which is regulated by protein phosphatase 2A (PP2A). Combining hyperthermia with HDAC inhibitors vorinostat or panobinostat leads to better anti-cancer effects compared to monotherapy. *KEAP1* and *DPP7* as genes affected by heat-induced inhibition of H3K9ac, and combining them with hyperthermia can better induce apoptosis in tumor cells. This study reveals previously unknown mechanisms of H3K9ac decreased by heat shock in cancer cells and highlights a potential combinational therapy involving hyperthermia and targeting of these new mechanisms.

## Introduction

Organisms can be exposed to different forms of stress, including sudden temperature increases. Heat shock, a common physiological and pathological stress, triggers an ancient signaling pathway that induces the transient expression of heat shock or heat stress proteins (HSPs) in cells 1, 2. Thermal stimulation, clinically known as hyperthermia treatment, is a low-cost, convenient, and safe adjunctive modality in clinical practice 3, 4. Notably, hyperthermia has been shown to exhibit maximum efficacy when combined with other established cancer therapies, such as radiotherapy or chemotherapy 5-8. Although hyperthermia therapy has emerged as a promising anti-tumor therapeutic modality, the efficacy of hyperthermia as a monotherapy is limited 5, 9. The effectiveness of hyperthermia therapy alone may be restricted in some cancer cells that exhibit resistance to heat-induced cell death, and the underlying mechanisms contributing to this phenomenon remain largely unexplored.

Histone modifications play a crucial role in regulating gene expression and chromatin structure 10, 11, and are highly sensitive to environmental factors, including heat stress 12, 13. Dysregulation of these modifications has been implicated in carcinogenesis 14, 15. One such modification, H3K9 acetylation (H3K9ac), is an epigenetic modification of the histone H3 protein, associated with gene transcription activation 16, 17. Targeting histone modifications is an emerging strategy for cancer therapy 18, 19. Several drugs that target histone modifying enzymes, particularly histone deacetylases (HDACs) that regulate histone acetylation levels 20, 21, have been developed and are being marketed, such as vorinostat (SAHA) and panobinostat (LBH589) 22-24. SAHA was approved by the US FDA for the treatment of T-cell lymphoma in 2006 25. Studies have also shown that it inhibits the activity of HDAC 1, HDAC 2, HDAC 3, and HDAC 6 enzymes, and it has some therapeutic effects in treating gastrointestinal cancer 26, 27. LBH589 is the first HDAC inhibitor approved by the US FDA for the treatment of multiple myeloma, which was launched in 2015 28, 29. It is a potent pan-deacetylase inhibitor that has demonstrated promising activity against hematologic and solid tumors in numerous research studies 30, 31. Therefore, targeting histone modifications is a promising approach to developing new therapies for cancer treatment.

However, little is known regarding heat stress's role in altering histone modifications. Our study revealed that heat shock suppresses H3K9ac via HDAC6 regulation, which involves disrupting the HDAC6 and heat shock protein 90 (HSP90) interaction, promoting HDAC6 nuclear localization, and increasing HDAC6 phosphorylation. Treatment with HDAC inhibitors SAHA and LBH589 could sensitize cancer cells to hyperthermia. We identified *KEAP1* and *DPP7* as genes controlled by H3K9ac in response to heat shock that can better induce apoptosis in tumor cells when combined with hyperthermia. Our study discovered new mechanisms of H3K9ac modulation by heat shock and suggested a possible therapy combining hyperthermia and targeting these mechanisms.

## Results

### Heat shock induces the downregulation of H3K9ac

**Figure 1:**
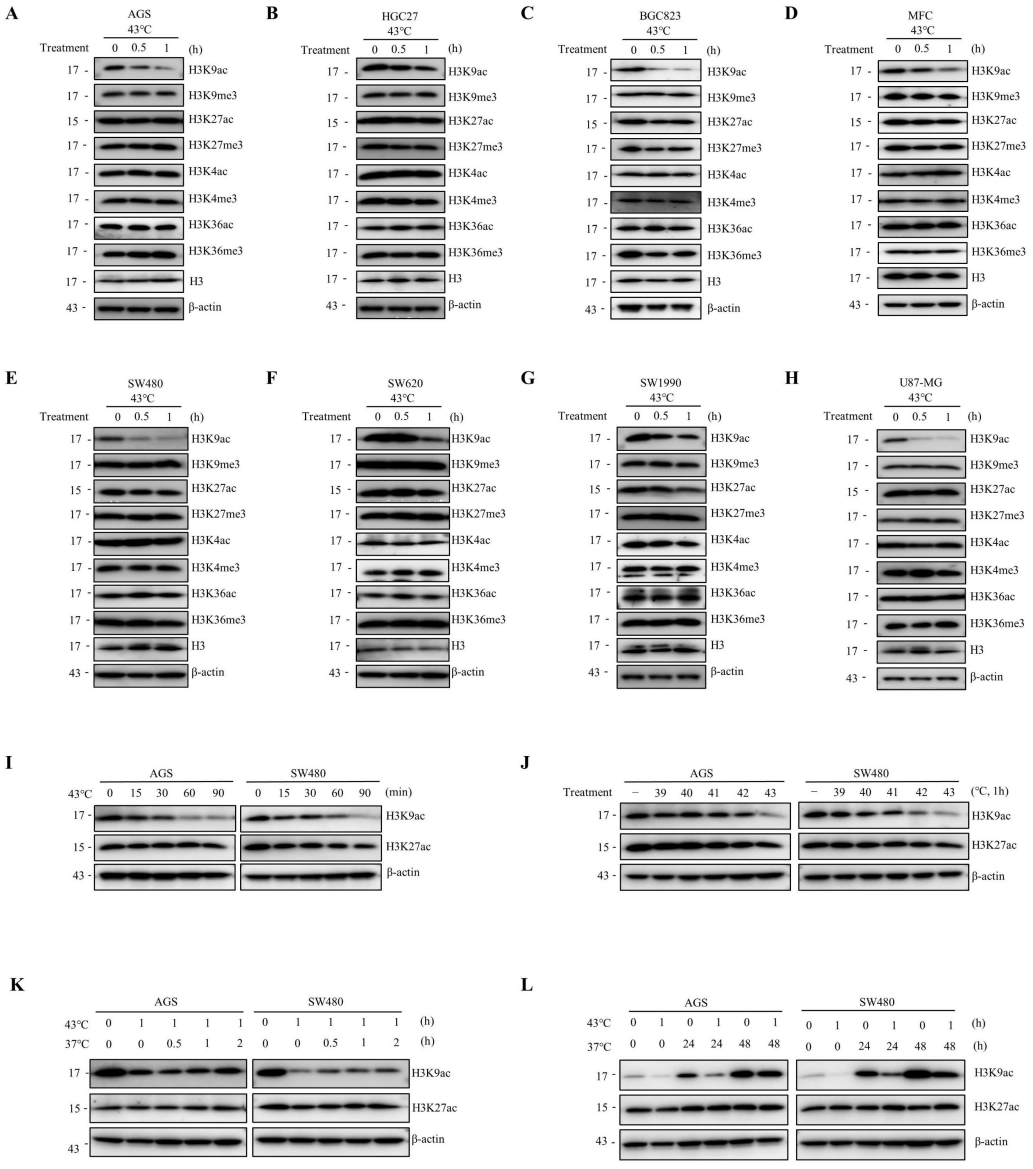
** Heat shock reduces H3K9ac. A-H** AGS (A), HGC27 (B), BGC823 (C); MFC (D), SW480 (E), SW620 (F), SW1990 (G), and U87-MG (H) cells were heat shocked at 43°C for 30 min or 1 hour, followed by immunoblotting to determine changes in acetylation and methylation at histone 3 lysine 4, 9, 27, and 36. **I** AGS and SW480 cells were exposed to heat shock at 43°C for the indicated times and analyzed by western blotting (WB) to assess the changes in acetylation at histone 3 lysine 9 and 27. **J** AGS and SW480 cells were subjected to heat shock at the indicated temperature for 1 hour and analyzed by WB to evaluate the changes in acetylation at histone 3 lysine 9 and 27. **K-L** AGS and SW480 cells were heat shocked at 43°C for 1 hour and allowed to recover at 37°C for various durations (0.5 hour, 1 hour, 2 hours in **K**; 24 hours and 48 hours in **L**) before cell lysates were collected for WB to determine changes in acetylation at histone 3 lysine 9 and 27.

To investigate the impact of heat shock on post-translational modifications of histone H3, we analyzed alterations in acetylation and methylation at H3K4, H3K9, H3K27, and H3K36. Our results indicate that heat shock induces a decrease in the specificity of H3K9ac across various cell lines, including human gastric cancer cells (AGS, HGC27, and BGC823), mouse gastric cancer cells (MFC), human intestinal cancer cells (SW480 and SW620), human pancreatic cancer cells (SW1990), and human glioma cells (U87-MG) (Fig. 1A-H). Notably, in AGS and SW480 cells, heat shock treatment at 43°C resulted in a significant downregulation of H3K9ac expression at 15 minutes, with a more pronounced decrease observed with increasing treatment duration (Fig. 1I). The kinetics of the decrease in H3K9ac varied among different cell types. Moreover, our findings demonstrated a significant H3K9ac downregulation at 43°C, exhibiting more pronounced effects compared to treatment at 39°C, 40°C, 41°C, and 42°C after a 1-hour exposure in multiple cancer cell lines (Fig. 1J and Fig. S1A). The decrease in H3K9ac was found to be significant when heat shock was applied for 1 hour at 43°C. Subsequently, we observed a partial restoration of the heat-induced downregulation of H3K9ac within a short period of 2 hours after returning the cells to 37°C and further culturing (Fig. 1K and Fig. S1B). However, over a longer period of 24 or 48 hours, the degree of restoration was more pronounced (Fig. 1L). Additionally, we found that fever conditions (39.5°C for 8h or 40°C for 6h) also resulted in a decrease in H3K9ac (Fig. S1C). These observations demonstrate that heat shock induces a rapid and significant downregulation of H3K9ac that is universal across different cell types.

### The downregulation of H3K9ac induced by heat shock is mainly regulated by HDAC6

**Figure 2:**
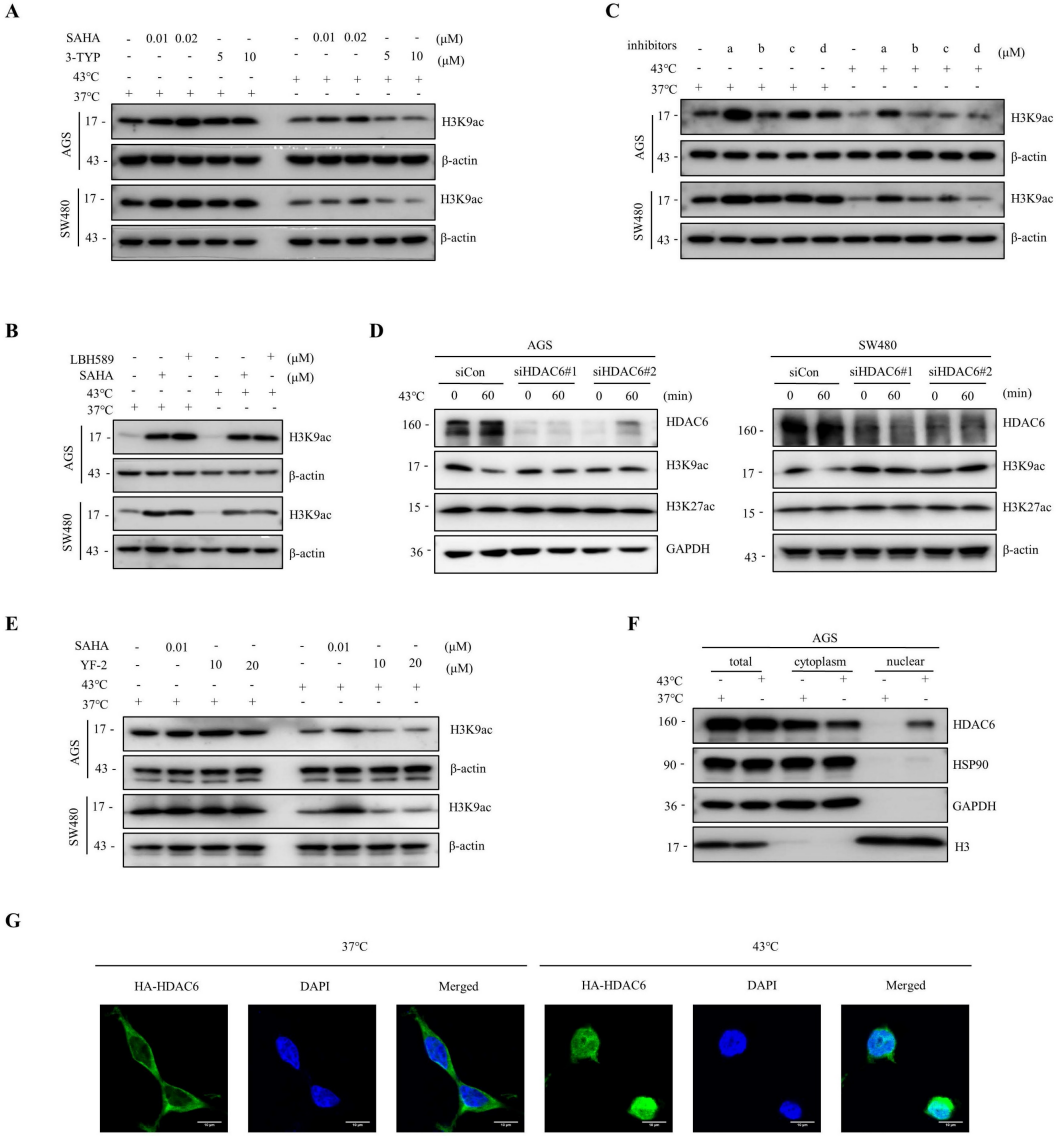
**Heat shock-induced H3K9ac downregulation is mainly influenced by HDAC6. A-B** AGS and SW480 cells were treated with SAHA (0.01 and 0.02 μM) and 3-TYP (5 and 10 μM) (A) or SAHA (0.1 μM) and LBH589 (0.05 μM) (B), followed by heat shock at 43°C for 1 hour. Changes in H3K9 acetylation were evaluated by WB. **C** AGS and SW480 cells were treated with a (0.5 μM), b (1 μM), c (0.5 μM), or d (1 μM), followed by heat shock at 43°C for 1 hour. Changes in H3K9 acetylation were analyzed by WB. a: ACY-241, b: TC-H 106, c: ACY-775, d: PCI34051. **D** AGS and SW480 cells were pre-transfected with control siRNA (siCon) or siRNAs targeting HDAC6 and subjected to heat shock at 43°C for 1 hour. Cells were lysed and evaluated by WB. Two independent siRNAs were used. **E** AGS and SW480 cells were treated with SAHA (0.01 μM) and YF-2 (10 and 20 μM), followed by heat shock at 43°C for 1 hour. Changes in H3K9ac were evaluated by WB. **F-G** AGS cells were fixed and subjected to Nuclear cytoplasmic fractionation assay and stained for WB (F) and immunofluorescence microscopy using an anti-HA antibody, in case of AGS cells were transfected with HA-HDAC6 plasmid (G). Scale bars, 10 μm.

Histone acetylation modifications are important epigenetics regulated by histone acetyltransferases (HATs) and HDACs 32, 33. To investigate whether HDACs are involved in the heat shock-induced decrease of H3K9ac, we screened major classes of HDAC inhibitors. Our findings showed that SAHA and LBH589, but not 3-TYP, a Sirtuins inhibitor, blocked the downregulation of H3K9ac by heat shock (Fig. 2A, B). Furthermore, we found that a selective HDAC6 inhibitor ACY-241, but not inhibitors of class I HDAC TC-H 106 and selective HDAC8 inhibitor PCI34051, also blocked the downregulation of H3K9ac by heat shock (Fig. 2C). Additionally, knockdown of HDAC6 by short interfering RNA (siRNA) also largely blocked the decrease of H3K9ac induced by heat shock (Fig. 2D). To investigate whether the function of HATs is inhibited under heat shock stress conditions, we employed the HATs activator, YF-2, to treat cells before subjecting them to heat shock. The results revealed that YF-2 could not block the heat shock-induced downregulation of H3K9ac, indicating that the function of HATs was not inhibited under heat shock stress (Fig. 2E).

We also investigated the changes in the expression of apoptosis-related proteins, such as c-PARP and c-Caspase 3, induced by HDAC inhibitors with or without heat shock at 43°C (Fig. S2A). To investigate the anti-cancer effects of the combination therapy of ACY-241 and hyperthermia, we analyzed the expression of c-PARP and c-Caspase 3 by Western blot analysis and performed CCK-8 assays and flow cytometry analysis. Our results demonstrated that under hyperthermia at 43°C, ACY-241 treatment led to a more significant induction of c-PARP and c-Caspase 3 expression in both AGS and SW480 cells (Fig. S2A). Furthermore, the combination of ACY-241 and hyperthermia significantly inhibited tumor cell growth and induced approximately twice as many apoptotic cells compared to ACY-241 alone (Fig. S2B-D). These observations indicate that HDAC6 plays a major role in the heat shock-induced H3K9ac downregulation and its potential implications for tumor cell apoptosis.

### Synergic effects of combining HDAC inhibitors and hyperthermia treatment on Apoptosis Activation

**Figure 3:**
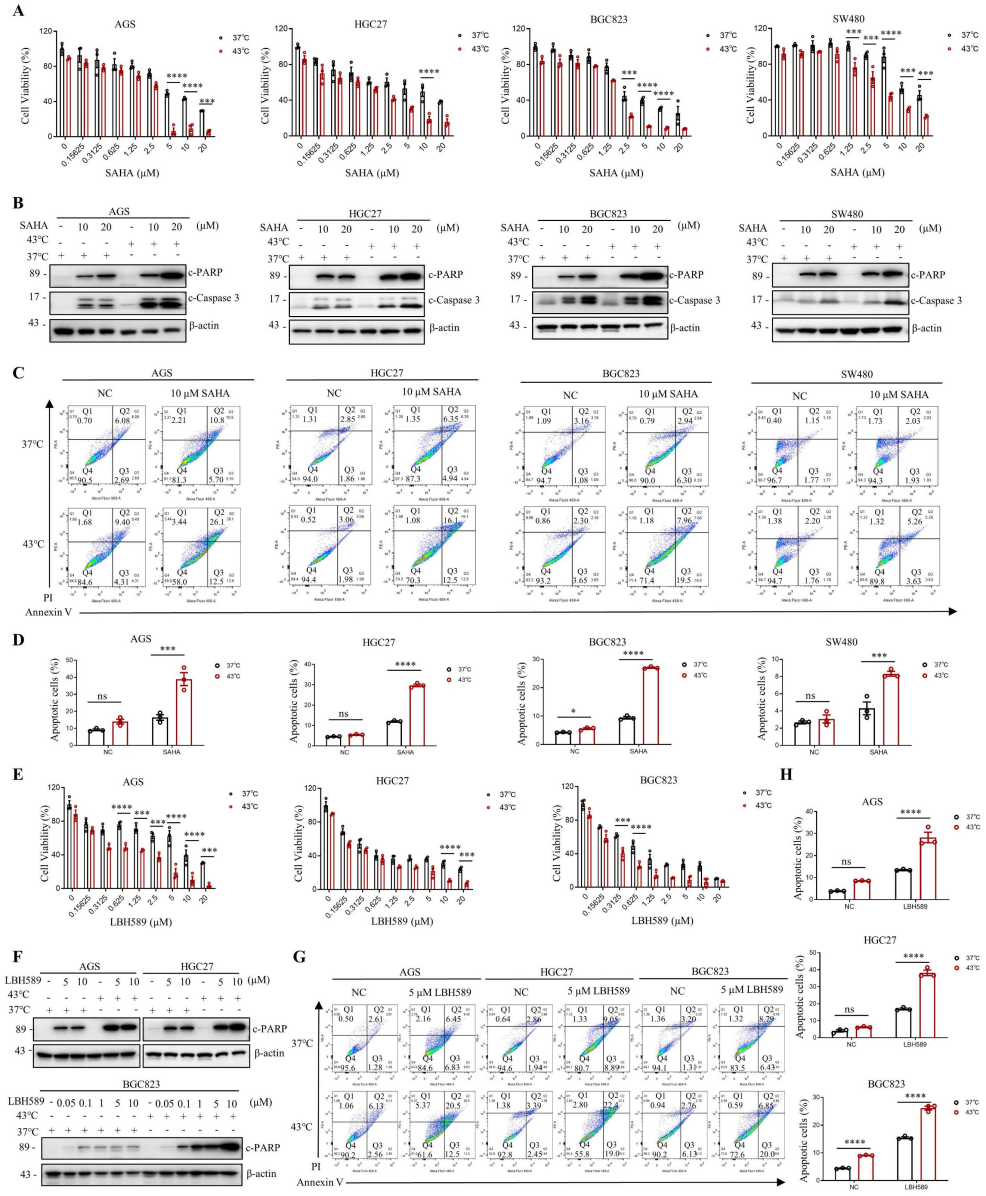
**SAHA and LBH589 enhance heat shock-induced apoptosis. A-D** Effect of SAHA on heat shock-induced apoptosis in AGS, HGC27, BGC823, and SW480 cells analyzed by CCK8 assay (A), WB (B), and flow cytometry assay (C). Prior to heat shock at 43°C for 1 h, cells were treated with SAHA as the indicated concentration for 24 h (B, C) or 48 h (A). Percentage of apoptotic cells was quantified by flow cytometry assay (D). N=3. **E-H** Effect of LBH589 on heat shock-induced apoptosis in AGS, HGC27, and BGC823 cells analyzed by CCK8 assay (E), WB (F), and flow cytometry assay (G). Prior to heat shock at 43°C for 1 h, cells were treated with LBH589 as indicated concentration for 24 h. Percentage of apoptotic cells was quantified by flow cytometry assay (H). N=3. Date: mean ± SEM. Statistical analysis: two-way ANOVA. *p-value <0.05, ***p-value <0.001, ****p-value <0.0001. ns, no significance.

Given the current lack of an HDAC6-specific inhibitor, we investigated the efficacy of marketed HDAC inhibitors SAHA and LBH589 in combination with hyperthermia. We have investigated the impact of the combination of hyperthermia and SAHA or LBH589 on apoptosis at different temperatures. The results show that the combination of SAHA or LBH589 with hyperthermia at 43°C can better induce tumor cell apoptosis and inhibit the growth of tumor cells (Fig. S2E-F). In gastrointestinal cancer cells, our results indicate that the combination of SAHA or LBH589 with hyperthermia is more effective in inhibiting tumor cell growth than the use of SAHA or LBH589 or heat therapy alone, as evidenced by the CCK8 and clone formation assays (Fig. 3A, E and Fig. S3A). Western blotting revealed that SAHA or LBH589 consistently increased the expression of apoptosis-related proteins, such as c-PARP and/or c-Caspase 3, particularly when used in combination with hyperthermia (Fig. 3B, F). Moreover, we observed that the expression of c-PARP and/or c-Caspase 3 was enhanced as the concentration of SAHA or LBH589 treatment increased in combination with hyperthermia, as compared to SAHA or LBH589 alone (Fig. 3F and Fig. S3D).

To further evaluate the functional synergy between HDAC inhibitors and heat shock on apoptosis activation, we used apoptosis-specific assay Annexin V to distinguish early and late apoptosis. Our results demonstrate that the combined treatment of SAHA with hyperthermia treatment induces a significant increase in both early and late apoptosis in cells compared to treatment with SAHA or hyperthermia alone (Fig. 3C and Fig. S3B). Similarly, the combined use of LBH589 resulted in comparable results (Fig. 3G and Fig. S3C). In particular, the combined treatment of SAHA and hyperthermia induced a significant increase in apoptosis in AGS, HGC27, BGC823, and SW480 cells compared to treatment with SAHA alone (2.4-fold, 2.5-fold, 2.9-fold, and 1.9-fold, respectively) (Fig. 3D). Likewise, the combined treatment of LBH589 and hyperthermia resulted in a significant increase in apoptosis in AGS, HGC27, and BGC823 cells compared to treatment with LBH589 alone (2.1-fold, 2.3-fold, 2.9-fold, and 1.7-fold, respectively) (Fig. 3H). Importantly, the synergistic effect observed was found to be independent of the ferroptosis marker GPX4 and the pyroptosis marker GSDMD, as the combined use of SAHA and heat therapy did not result in an increase in their expression levels (Fig. S3E).

It is noteworthy that SAHA has been approved by the FDA for the treatment of lymphoma. Our results demonstrate that heat shock downregulates H3K9ac in lymphoma cells (Fig. S1D), and that the combined treatment of SAHA and heat shock induces better apoptosis induction in lymphoma cells than treatment with SAHA or heat shock alone, as shown by the CCK8 assay (Fig. S3F) and flow cytometry assays (Fig. S3G-H).

### Heat shock induces the suppression of HDAC6-HSP90 interaction, enhancing phosphorylation and nuclear accumulation of HDAC6

**Figure 4:**
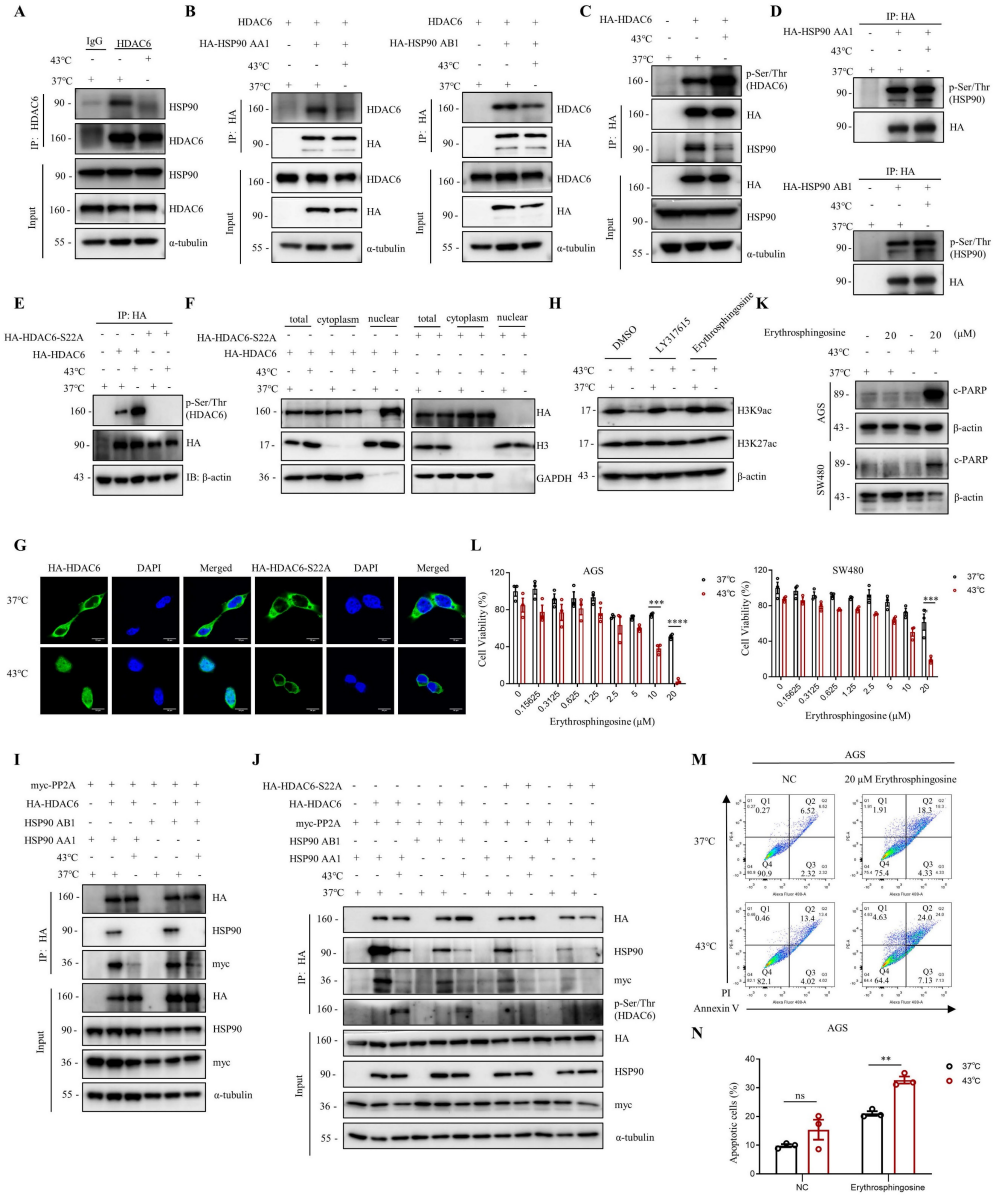
** Heat shock suppresses the HDAC6-HSP90 interaction, increases HDAC6 phosphorylation, and enhances nuclear localization. A** Co-immunoprecipitation (Co-IP) of HSP90 was detected by WB in AGS cells after heat shock at 43°C for 1 h and HDAC6 immunoprecipitation (IP). **B** Co-IP of HDAC6 and HA-HSP90 was detected by WB in HEK293T cells co-transfected with plasmids for HDAC6 and HA-HSP90 AA1 or HA-HSP90 AB1 and subjected to heat shock at 43°C for 1 h. **C-E** HDAC6 phosphorylation and Co-IP of HSP90 were detected by WB. HEK293T cells were transfected with HA-HDAC6 plasmids and subjected to heat shock. HDAC6 was immunoprecipitated with HA antibody. Phosphorylation of HDAC6 was detected using Pan Phospho-Serine/Threonine antibody (C). Similar experiments were performed with HA-HSP90 AA1 or HA-HSP90 AB1 plasmids (D), and with HA-HDAC6-S22A plasmid (E). **F-G** Nuclear localization was detected in cells. HEK293T cells were co-transfected with HA-HDAC6-S22A and HA-HDAC6 plasmids, followed by heat shock for 1 h. Nuclear cytoplasmic fractionation assay was performed to and analyzed by WB (F). Immunofluorescence microscopy with an anti-HA antibody for AGS cells were transfected with HA-HDAC6-S22A and HA-HDAC6 plasmid (G). **H** Changes in acetylation at histone 3 lysine 9 and 27 were determined by immunoblotting in AGS cells were treated with LY317615 (5 μM) and Erythrosphingosine (5 μM) for 2 h before heat shock. **I-J** Co-precipitated proteins were detected by WB using anti-HA antibody after IP in HEK293T cells transfected with indicated plasmids and subjected to heat shock. **K-N** Effect of Erythrosphingosine on heat shock-induced apoptosis in AGS and/or SW480 cells analyzed by WB (K), CCK8 assay (L) and flow cytometry assay (M). Before treatment, cells were treated with Erythrosphingosine as indicated concentration for 24 h (K, M) or 48 h (L). Percentage of apoptotic cells was quantified by flow cytometry assay (N). N=3. Date: mean ± SEM. Statistical analysis: two-way ANOVA. **p-value <0.01, ***p-value <0.001, ****p-value <0.0001. ns, no significance.

The activity of HDAC6 is influenced by various factors, including interactions with other proteins, post-translational modifications, and localization 34-36. Herein, we investigated how HDAC6 regulates the heat shock-induced downregulation of H3K9ac. We found that HDAC6 nuclear localization significantly increased under heat shock conditions at 43°C, as demonstrated by the nuclear extract assay (Fig. 2F, 4F and Fig. S4B) and immunofluorescence staining (Fig. 2G). Besides histones, HDAC6 has been reported to deacetylate various nonhistone proteins, including Hsp90, a molecular chaperone that plays a vital role in heat stress response 37. Our immunoprecipitation assay showed that heat shock decreases HDAC6 and HSP90 interaction (Fig. 4A). Further co-immunoprecipitation experiments revealed that heat shock inhibits the interaction between HDAC6 and either HSP90 AA1 or HSP90 AB1 (Fig. 4B).

Our experiments showed that heat shock increased HDAC6 phosphorylation (Fig. 4C), whereas HSP90 AA1 and HSP90 AB1 phosphorylation remained unchanged (Fig. 4D). It has been reported that the activity of HDAC6 deacetylase increases after its phosphorylation at serine 22 (phospho-HDAC6) 38. Consistent with this, our experiments showed that heat shock induces HDAC6 serine-22 phosphorylation (Fig. 4E). Moreover, we investigated whether phosphorylation of HDAC6 at serine-22 regulates heat-induced nuclear translocation of HDAC6. Mutation of the serine residue at the phosphorylation site to alanine or glutamate, as demonstrated by the nuclear extract assay (Fig. 4F and Fig. S4B) and immunofluorescence (Fig. 4G), revealed that heat shock could not induce an increase in HDAC6 nuclear localization. Therefore, these findings indicate that heat-induced HDAC6 nuclear translocation is regulated by phosphorylation at serine-22.

Furthermore, we observed that Erythrosphingosine, a PP2A activator that inhibits PKC, can reverse the downregulation of H3K9ac induced by heat shock, while LY317615, a PKC inhibitor, did not (Fig. 4H). Our co-immunoprecipitation assay showed that HDAC6 interacts with PP2A, and heat shock inhibits the interaction between HDAC6 and PP2A (Fig. 4I and Fig. S4C). Subsequently, we investigated whether phosphorylation of HDAC6 affects its interaction with HSP90 and PP2A. The experimental findings demonstrated that upon mutation of the serine residue at the phosphorylation site to alanine of HDAC6, its interaction with HSP90 was weakened. Similarly, the interaction between HDAC6 and PP2A also showed a reduced affinity (Fig. 4J). Based on these findings, we investigated the anti-cancer effects of combination treatment with Erythrosphingosine and hyperthermia. Our CCK8 assay demonstrated that the combination treatment had a more significant inhibitory effect on tumor cells than either treatment alone (Fig. 4L). Furthermore, Western blot analysis of the apoptotic marker c-PARP (Fig. 4K) and flow cytometry experiments (Fig. 4M, N and Fig. S4D) showed that the combination treatment induced greater tumor cell apoptosis. These results suggest that PP2A may play a role in regulating the expression of hyperthermia-enhanced HDAC6 phosphorylation.

### Gene Regulation through the downregulation of H3K9ac induced by heat shock

**Figure 5:**
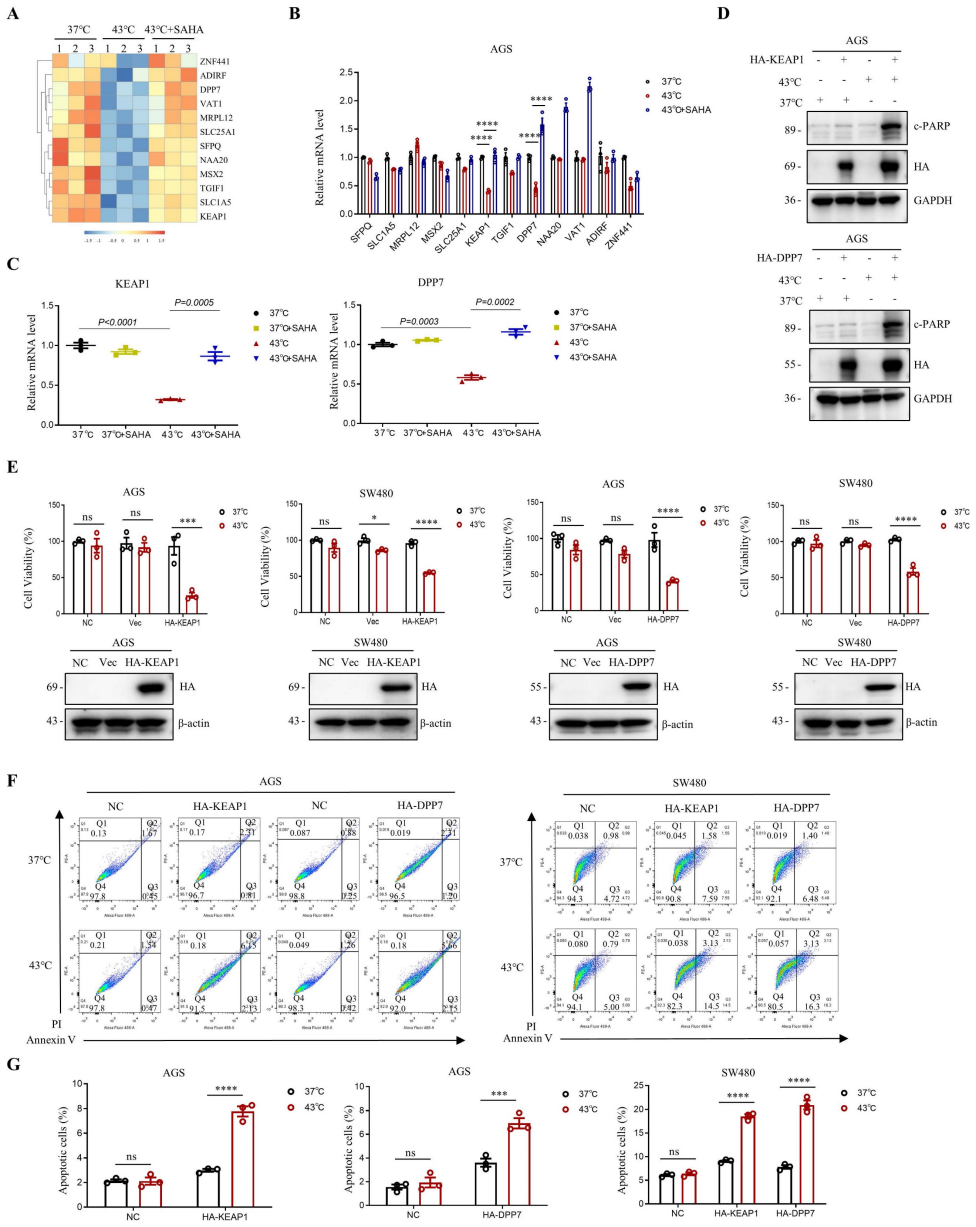
** Modulation of gene expression via H3K9ac reduction induced by heat shock. A** Heat map displaying representative genes that were downregulated by heat shock at 43°C for 1 h and subsequently upregulated by SAHA treatment (0.02 μM) in AGS cells. N=3.** B** Real-time PCR analysis of genes highlighted in A that showed significant differential expression with a *p*-value <0.0001. These genes were downregulated by heat shock at 43°C for 1 hour and subsequently upregulated by SAHA (0.02 μM). N=3.** C** Changes in mRNA expression levels of KEAP1 and DPP7 were determined by real-time PCR. AGS cells were treated with heat shock at 43°C for 1 h in the presence or absence of SAHA (0.02 μM). N=3.** D-G** AGS and/or SW480 cells were transfected with plasmids HA-KEAP1 or HA-DPP7, followed by treatment with or without heat shock at 43°C for 1 h. WB (D), CCK8 assay (E) and flow cytometry (F) were used for detection. Percentage of apoptotic cells was quantified by flow cytometry assay (G). N=3. Date: mean ± SEM. Statistical analysis: The data in C was using two-tailed unpaired t test; the others were tested with two-way ANOVA. *p-value <0.05, ***p-value <0.001, ****p-value <0.0001. ns, no significance.

A decrease in H3K9ac levels in tumor cells has been shown to play a role in the repression of genes 17, 39. In this study, we investigated the effect of downregulation of H3K9ac induced by heat shock on gene expression in AGS cells, using RNA-seq analysis. We found that heat shock at 43°C resulted in the upregulation of 792 genes and the downregulation of 292 genes in AGS cells (Fig. S5A, B). Among the genes that were downregulated by heat shock, we screened for those that could be upregulated by SAHA in the heat-shocked 43°C + SAHA group (Fig. 5A). Further detection using RT-PCR revealed that *KEAP1* and *DPP7* were better able to be simultaneously up-regulated by SAHA after being down-regulated by heat shock at 43°C (Fig. 5B, C and Fig. S5C).

To investigate the combined effects of KEAP1 and DPP7 with heat shock treatment, we overexpressed KEAP1 or DPP7 in AGS and SW480 cells, followed by heat shock treatment. Western blot analysis revealed that overexpression of KEAP1 or DPP7 led to a significant increase in c-PARP levels when followed by hyperthermia. (Fig. 5D). Moreover, we observed a significant reduction in cell viability in AGS and SW480 cells when KEAP1 or DPP7 overexpression was combined with hyperthermia, as compared to either heat treatment alone or overexpression of KEAP1 or DPP7 alone (Fig. 5E). Furthermore, flow cytometry assays showed that the combination of hyperthermia and overexpression of KEAP1 or DPP7 resulted in a significant increase in apoptotic cell death in AGS cells compared to overexpression of KEAP1 or DPP7 alone (2.6-fold and 1.9-fold, respectively). Likewise, in SW480 cells, hyperthermia treatment combined with overexpression of KEAP1 or DPP7 also showed a significant increase in apoptosis when compared to treatment with overexpression of KEAP1 or DPP7 alone (2-fold, 2.7-fold respectively) (Fig. 5F, G and Fig. S5D). Our results suggest that the combination of KEAP1 and DPP7 with hyperthermia treatment can more effectively inhibit tumor cell growth and induce tumor cell apoptosis.

### Combination treatment of SAHA or LBH589 with hyperthermia has great inhibitory effects on gastric cells growth *in vivo*

**Figure 6:**
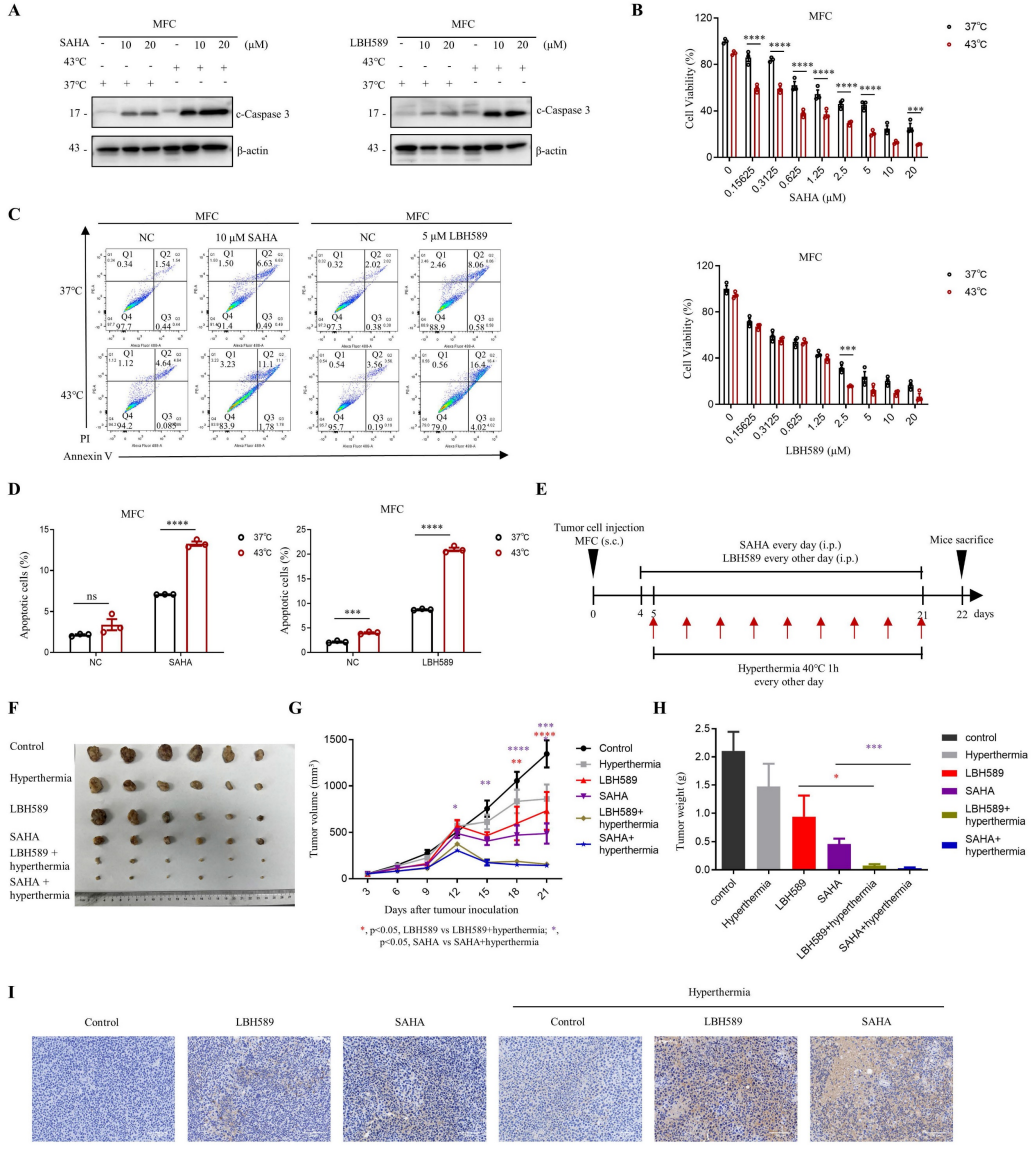
** Synergistic effects of combining SAHA or LBH589 with hyperthermia on gastric cancer cells growth *in vivo*. A-D** MFC cells were treated with or without heat shock at 43°C for 1 hour and were then detected for WB (A), CCK8 assay (B) and flow cytometry (C) after pre-treatment with SAHA at the indicated concentration for 24 h (A, C) or 48 h (B) and LBH589 for 24 h (A-C). Percentage of apoptotic cells was quantified by flow cytometry assay (D). N=3. **E** Schedule of animal experiments. 615 mice were subcutaneously (s.c.) injected with MFC cells. SAHA (25 mg/kg body weight) and LBH589 (10 mg/kg body weight) were administered via intraperitoneal (i.p.) injection every day or every other day, respectively, starting on day 4 after cell injection. Mice were exposed to hyperthermia at 40°C for 1 hour every other day starting on day 5 after cell injection. The mice were sacrificed and the tumors were collected on day 22. **F** Representative images MFC tumor burdens in mice. N=6. **G** Tumor growth was monitored at the indicated times. N=6. **H** Tumor weights of MFC tumors on day 22. N=6. **I** Immunohistochemistry of cleaved caspase-3. Scale bar, 100 μm. Date: mean ± SEM. Statistical analysis: The data in H was analyzed using two-tailed unpaired t test; the others were analyzed using two-way ANOVA. *p-value <0.05, **p-value <0.01, ***p-value <0.001, ****p-value <0.0001. ns, no significance.

We evaluated the efficacy of combined treatment with SAHA or LBH589 and hyperthermia on tumor growth using murine syngeneic models. Our study revealed that the combination of SAHA or LBH589 with hyperthermia showed a stronger expression of c-Caspase 3 in western blot (Fig. 6A) and lower cell viability in the CCK8 assay (Fig. 6B) in MFC cells compared to their individual effects. Further analysis using flow cytometry indicated that the combination of hyperthermia with SAHA or LBH589 induced more significant total cell apoptosis than SAHA or LBH589 alone (Fig. 6C, D and Fig. S6A).

To evaluate the *in vivo* efficacy of the combination treatment, we inoculated MFC cells in 615 mice and monitored tumor growth until day 22 (Fig. 6E). Our results indicated that H3K9ac is specifically downregulated under the hyperthermia condition used in the mouse models (Fig. S6B). And the results also demonstrated that the combination treatment of SAHA or LBH589 with hyperthermia significantly suppressed tumor growth compared to their individual effects (Fig. 6F-H). Specifically, the tumor volume and weight of mice treated with SAHA + hyperthermia decreased by approximately 3.4-fold and 6-fold, respectively, compared to those treated with SAHA alone. Similarly, the tumor volume and weight of mice treated with LBH589 + hyperthermia decreased by approximately 4.6-fold and 12-fold, respectively, compared to those treated with LBH589 alone (Fig. 6G, H). Immunohistochemical staining revealed a notable elevation of cleaved caspase-3, an apoptosis marker, in the SAHA or LBH589 combined with hyperthermia treatment group when compared to the groups subjected to either SAHA or LBH589 alone, or hyperthermia alone (Fig. 6I). Collectively, our findings suggest that the combination treatment of SAHA or LBH589 with hyperthermia can effectively inhibit the growth of gastric tumors *in vivo*.

## Discussion

In this study, we discovered that heat shock could significantly decrease the level of H3K9ac in various cell types and diverse conditions. We observed that the downregulation of H3K9ac induced by heat shock conditions at 43°C is a rapid, sustained and reversible process, resembling the self-protective mechanism of the organism under stress, commonly known as the "heat shock response".

Our findings indicate that heat shock primarily downregulates H3K9ac through the regulation of HDAC6, although the involvement of other HDACs cannot be entirely excluded. This conclusion is supported by the recovery effects of HDAC inhibitors and the downregulation of H3K9ac upon heat shock-induced knockdown of HDAC6. While the HDAC6-specific inhibitor, ACY-241, and HDAC6 knockdown can effectively restore the downregulation of H3K9ac induced by heat shock, the recovery effect of another selective HDAC6 inhibitor, ACY-775, is not obvious. It is speculated that ACY-775 itself may not function well as an inhibitor under these conditions. Our subsequent experiments indicate that the combined use of hyperthermia and ACY-241 leads to better suppression of tumor cell growth and induction of tumor cell apoptosis compared to their individual effects. This finding indirectly supports the hypothesis that heat-induced inhibition of H3K9ac is mainly regulated by HDAC6 and provides new theoretical evidence for combining hyperthermia with HDAC inhibitors.

HDAC6 is a unique deacetylase primarily localized in the cytoplasm and possesses a robust ability to deacetylate not only histones but also nonhistone proteins 40-42. We have observed an increase in the nuclear translocation of HDAC6 under heat shock. This observation, combined with the results indicating that HDAC6 predominantly regulates the downregulation of H3K9ac under heat shock (Fig. 2), suggests that HDAC6 may efficiently enter the nucleus and exert its deacetylation function on H3K9ac. We observed that heat shock could inhibit the interaction between HSP90 and HDAC6. Previous studies have shown that blocking the interaction between HDAC6 and Hsp90 plays a crucial regulatory role in a wide range of diseases, especially in cancer 37, 43. We observed that heat shock could increase the nuclear translocation and phosphorylation of HDAC6, which is regulated by phosphorylation at serine-22. Therefore, we hypothesize that heat-induced dissociation of the HDAC6-HSP90 complex results in increased levels of free HDAC6, facilitating its translocation into the nucleus. In addition, the increase in HDAC6 phosphorylation enhances its nuclear entry, and its entry is regulated by phosphorylation at serine-22. This suggests that the increased activity of HDAC6 in the nucleus is mainly in the form of phosphorylation, which enhances its deacetylation of histones and better explains the downregulation of H3K9ac induced by heat, which is mainly regulated by HDAC6. However, whether HDAC6 enters the nucleus in a phosphorylated form or is phosphorylated after nuclear entry requires further investigation. Additionally, we observed that the increased phosphorylation of HDAC6 may be regulated by the phosphatase PP2A. Activators of PP2A in combination with hyperthermia also have a synergistic effect, providing new theoretical evidence for the combined use of hyperthermia and chemotherapy in the clinic from another perspective.

Based on the mechanism of H3K9ac downregulation induced by heat shock described above, this study elucidates the potential limitations of hyperthermia monotherapy, which may guide the development of subsequent combination therapies in clinical practice. Gastric cancer, a disease with high incidence and mortality rates, is currently a research priority in oncology 44, 45, and improving treatment efficacy and patient outcomes is crucial for enhancing the quality of life and overall survival of gastric cancer patients. Therefore, hyperthermia was combined with the marketed HDAC inhibitors SAHA and LBH589 in gastric cancer cells, and the results showed that the combined application of hyperthermia with SAHA or LBH589 exhibits superior anticancer effects when compared to their respective individual usage. Previous studies have shown that SAHA exhibits potent cytotoxicity against gastric cancer cells *in vitro* 46, 47. However, its efficacy in treating patients was not demonstrated in a phase II trial, which may be attributed to the dose-limiting toxicities of SAHA 46. The combination of hyperthermia and SAHA may provide a promising new approach to the treatment of gastric cancer in the clinic. By using relatively low doses of SAHA in combination with hyperthermia to achieve the same efficacy, the toxic effects caused by high doses of SAHA can be avoided, and the therapeutic effect of SAHA and hyperthermia against gastric cancer can be fully realized. Currently, there is a limited number of studies exploring the role and mechanism of LBH589 in treating gastric cancer 48, 49, and a research gap exists regarding the efficacy of LBH589 in treating gastric cancer. Therefore, the combination of LBH589 and hyperthermia in this study may provide a new approach to the treatment of gastric cancer with LBH589 in clinical practice.

H3K9ac is a histone modification that is enriched in the promoter regions of transcriptionally active genes 39. Previous studies have shown that the combined use of hyperthermia and gene therapy can result in improved anti-cancer efficacy 50-52. In this study, we identified two genes, *KEAP1* and *DPP7*, that showed significant tumor growth delay and enhanced tumor cell apoptosis only when overexpressed in combination with hyperthermia. The role of KEAP1 in cancer is that it serves as a critical antioxidant response element, affecting cellular growth, proliferation, and invasion in cancer 53, 54. DPP7 is involved in the regulation of various biological processes in tumors, including cell apoptosis, proliferation, invasion, and metastasis, and may play a tumor-suppressive role in certain types of tumors 55, 56. Currently, there is a controversy regarding whether KEAP1 and DPP7 can act as tumor suppressors. However, experiments conducted under hyperthermia, downregulation of H3K9ac results in decreased expression of KEAP1 and DPP7. Based on this, overexpression of *KEAP1* and *DPP7* under hyperthermia can overcome the limitations of using hyperthermia alone and enhance the anticancer efficacy of hyperthermia. Further research is required to explore the commonalities between the genes regulated by the downregulation of H3K9ac induced by heat shock. Based on the mechanism of gene regulation through the downregulation of H3K9ac induced by heat shock, this study provides a new theoretical basis for the combined use of hyperthermia and gene therapy.

This study demonstrates that blocking the reduction of H3K9 acetylation induced by heat shock effectively inhibits the growth of cancer cells under hyperthermic conditions, providing potential for improving the clinical efficacy of hyperthermia therapy in cancer by targeting this mechanism. The findings of this study provide a theoretical foundation and fresh perspectives for the development of new cancer treatments.

## Materials and Methods

### Cell culture

All of the cell lines were cultured at 37°C in a humidified 5% CO2 atmosphere. AGS, HGC27, BGC823, SW480, SW620, U87-MG and HEK293T were cultured in Dulbecco's modified Eagle's medium (DMEM) (Gibco™, Thermo Fisher Scientific, Waltham, MA USA), and SW1990 and MFC were cultured in RPMI-1640 medium (Gibco™, Thermo Fisher Scientific) containing 10% Fetal Bovine Serum (FBS) and 1% penicillin/streptomycin (Gibco™, Thermo Fisher Scientific). All the cell lines were from ATCC and routinely tested for mycoplasma contamination.

### Heat stress exposure

For all of the *in vitro* heat shock experiments, cells were placed in a culture incubator with an environmental temperature at 43°C and humidified 5% CO2 for indicated time periods.

For all of the *in vivo* heat shock experiments, male mice were housed in standard cages with ad libitum access to food and water and maintained at an ambient temperature of 25°C. Heat stress was induced by placing mice in a climate chamber maintained at 40°C for 1 hour. After the heat shock, mice were immediately returned to their original condition with an environmental temperature at 25°C and free access to food and water. The sham control mice underwent the same procedure without heat stress.

### Plasmids and Transfection

Plasmids pcmv-HA-HSP90 AA1 and pcmv-HA-HSP90 AB1 were kind gifts from Prof. Jianfeng Chen at the University of Chinese Academy of Sciences. The plasmid HDAC6 was a kind gift from Prof. Muqing Cao at Shanghai Jiao Tong University College of Basic Medical Science. Plasmids pcmv-HA-HDAC6, pcmv-HA-HDAC6-S22A, pcmv-HA-HDAC6-S22E, pcmv-myc-PP2A, pcmv-HA-KEAP1 and pcmv-HA-DPP7 were constructed using PCR. Duplex siRNAs targeting human HDAC6 were purchased from BioSune Biotechnology. Cells were transfected with lipofectamine 6000 (Beyotime Biotechnology, Shanghai, China) according to the instructions of the manufacturer.

The sequences were as follows: siCon (UUCUCCGAACGUGUCACGUTT); human siHDAC6#1 (CCAAUCUAGCGGAGGUAAA); siHDAC6#2 (GGAUGGAUCUGAACCUUGA).

### Antibodies and compounds

Antibodies against the following antigens were used: H3K9ac (PTM BIO, Hangzhou, China; Cat# PTM-112), H3K9me3 (PTM BIO; Cat# PTM-616), H3K27ac (PTM BIO; Cat# PTM-116), H3K27me3 (PTM BIO; Cat# PTM-5002), H3K4ac (PTM BIO; Cat# PTM-168), H3K4me3 (PTM BIO; Cat# PTM-613), H3K36me3 (PTM BIO; Cat# PTM-625), H3 (PTM BIO; Cat# PTM-1002), H3K36ac (Cell Signaling Technology, Danvers, MA USA; Cat# 27683S), HSP90 (Cell Signaling Technology; Cat# 4877S), HDAC6 (Cell Signaling Technology; Cat# 7558S), Cleave PARP (Cell Signaling Technology; Cat# 5625S), Cleave Caspase-3 (Cell Signaling Technology; Cat# 9664S), HA (ABclonal Technology, Wuhan, China; Cat# AE008), Pan Phospho-Serine/Threonine (Abmart, Shanghai, China; Cat# T91067), myc (Sigma-Aldrich, Saint Louis, USA; Cat# C3956), GSDMD (ABclonal Technology; Cat# A17308), GPX4 (Abways Technology, Shanghai, China; Cat# CY6959), β-actin (Cell Signaling Technology; Cat# 12620), GAPDH (Cell Signaling Technology; Cat# 8884), α-tubulin (Cell Signaling Technology; Cat# 9099). The following compounds were used: Vorinostat (SAHA) (Topscience, Shanghai, China; Cat# T1583), 3-TYP (Sellect, Shanghai, China; Cat# S8628), Citarinostat (ACY-241) (Topscience; Cat# T3661), Pimelic Diphenylamide 106 (TC-H 106) (Topscience; Cat# T3193), ACY-775 (Topscience; Cat# TQ0074), PCI34051 (Topscience; Cat# T6325), Panobinostat (LBH589) (Topscience; Cat# T2383), Erythrosphingosine (Topscience; Cat# T5891), LY317615 (Sellect; Cat# S1055), YF-2 (Sellect; Cat# S0022).

### Western Blotting

Cell lysates were boiled in sodium dodecyl sulfate (SDS) sample loading buffer, resolved by SDS-polyacrylamide gel electrophoresis (SDS-PAGE) and transferred to 0.45 μm PVDF membrane. The membranes were blocked in 5% milk in Tris-buffered saline and Tween 20 (TBST; 10 mM Tris-HCl [pH 8.0], 150 mM NaCl, 0.05% Tween 20) for 2 h at room temperature. Afterward, the membranes were incubated with antibodies as indicated in TBST overnight at 4°C and then washed with TBST three times at room temperature, probed with horseradish peroxide-linked anti-immunoglobulin for 1 h and washed three times with TBST again. Finally, immunoreactive products were visualized using enhanced chemiluminescence reagents and autoradiography.

### CCK8 assay

Cell viability was assessed by Cell Counting Kit-8 (Yeasen Biotechnology, Shanghai, China) according to the manufacturer's protocol. A total of 50 μL CCK8 was added per 1 mL medium and cultured for an additional 1.5 h. Then, the absorbance at 450 nm was measured by Gen5 Microplate Reader (BioTek Instruments, Vermont, USA) using the area scanning method (8×12 points for a 96 well plate). The results were calculated by GraphPad Prism 8.0 software.

### Colony-formation assay

Cells were seeded into 6-well plates at a density of 1000 cells per well and treated with the indicated condition. The medium was replaced every 6 days. After 12-14 days, the cells were fixed with 4% paraformaldehyde, stained with 0.1% crystal violet and washed twice with PBS.

### Apoptosis determination by flow cytometry

The PE Annexin V Apoptosis Detection Kit I (ShareBio, Shanghai, China) was used following the manufacturer's protocol. All samples were then processed using the Flow Cytometer (BD LSR Fortessa, BD Biosciences, New Jersey, USA). Cells undergoing early apoptosis (Q3) were characterized as Annexin V-positive and propidium iodide-negative cells, while cells undergoing late apoptosis (Q2) were characterized as Annexin V and propidium iodide double-positive cells. The results were analyzed using FlowJo version 10 software.

### Immunoprecipitation

Cells were harvested in lysis buffer (10 mM Tris [pH 7.5], 150 mM NaCl, 1% Triton X-100, 5 mM EDTA, containing protease and phosphatase inhibitors). The lysate was centrifuged at 13 000 rpm for 15 min at 4°C, and a small portion of the resulting supernatant was transferred as a control and boiled with SDS loading buffer. The remaining supernatants were incubated with corresponding antibodies with rotation overnight at 4°C. And then pulled down with protein A/G-Sepharose for an additional 3 h. After being washed with lysis buffer, the beads were boiled in SDS sample loading buffer and assessed by WB.

### Nuclear cytoplasmic fractionation assay

Nuclear cytoplasmic fractionation was prepared using NE-PER^TM^ Nuclear and Cytoplasmic Extraction Reagents (Thermo Fisher Scientific) in according with the manufacturer's instructions. Proteins from cell lysates or nuclear extracts were separated and assessed by WB. The cellular nuclear content was detected using H3 antibody, while the cytoplasmic content was detected using GAPDH antibody.

### Immunofluorescence

Cells were incubated on glass slides and permeabilized with 0.05% Triton X-100 in PBS for 12 min at 4°C. They were then blocked with 1% fetal bovine serum in PBS for 30 min at room temperature and incubated with corresponding primary antibodies at 4°C for 3 h. Alexa Fluor 488-labeled Goat Anti-Mouse IgG (1:200, Beyotime Biotechnology) or Alexa Fluor 647-labeled Goat Anti-Rabbit IgG (1:200, Beyotime Biotechnology) were used to pull down the primary antibodies at room temperature for 1 h. To stain the nuclei, the cells were co-stained with 4′,6-diamidino-2-phenylindole (DAPI) (1:10000, Thermo Fisher Scientific). The images were captured and analyzed by a Ti-E+A1 SI confocal laser scanning microscope system (Nikon, Tokyo, Japan).

### RNA isolation and real-time PCR

Total RNA from cells by using Total RNA Extractor Kit (Sangon Biotech, Shanghai, China) according to the manufacturer's instruction. In total, 1 μg RNA was reversed transcribed to cDNA using a ABScript III RT Master Mix for qPCR kit (ABclonal) and quantified by real-time PCR (RT-PCR) using ABclonal Genious 2X SYBR Green Fast qPCR Mix (No ROX) (ABclonal) in LightCycler96 instrument. The relative messenger RNA abundance of the target gene was calculated using the ΔΔCt method normalized to the expression of GAPDH.

The primers used (forward and reverse, respectively) were as follows: human *SFPQ* (5′-CAGCATGGCACGTTTGAGTA-3′ and 5′-CCTCCTCTTGCCTCAATTGC-3′); human *SLC1A5* (5′-TCGTGGAGATGGAGGATGTG-3′ and 5′-GATGAAACGGCTGATGTGCT-3′); human *MRPL12* (5′-CCTCAACGAGCTCCTGAAGA-3′ and 5′-GCTTTGATTTCCTGGGGCAG-3′); human *MSX2* (5′-ATATGAGCCCTACCACCTGC-3′ and 5′-GGGAAAGGGAGACTGAAGCT-3′); human *SLC25A1* (5′-AGCCCATGAACCCTCTGATC-3′ and 5′-TCCACACTTTGTTGAGCAGC-3′); human *KEAP1* (5′-CTCATCCAGCCCTGTCTTCA-3′ and 5′-CCAATCTGCTCAGCGAAGTT-3′); human *TGIF1* (5′-CTTTCTTCATCCGCTGGCTC-3′ and 5′-TCCACAGAGCTCGTTTCAGA-3′); human *DPP7* (5′-CCAGCAACAATGTGACCGAT-3′ and 5′-CAGGATCTTCTGGGTGGGAG-3′); human *NAA20* (5′-CAGAATTTCGACGCCTTGGT-3′ and 5′-CAATGTCTTCAGGCCTCACAG-3′); human *VAT1* (5′-TGTCCGACGAGAGAGAGGTA-3′ and 5′-CCATGAGGTCTGCGAAGTTG-3′); human *ADIRF* (5′-GCAACAGGTGGAGGGGAC-3′ and 5′-CCCAGAGAAGGTGTCAGAGG-3′); human *ZNF441* (5′-ATAGTCAATGTGGAGGACCCTT-3′ and 5′-AGATGAACGACCCATGAGGAC-3′); human *GAPDH* (5′-GGATTTGGTCGTATTGGGCG-3′ and 5′-TGACAAGCTTCCCGTTCTCA-3′).

### RNA-seq and bioinformatics analysis

Total RNAs were extracted from AGS cells using TRIzol (Thermo Fisher Scientific). The cells were divided into three groups: (1) untreated control, (2) subjected to heat shock at 43°C for 1 hour, and (3) treated with 0.02 μM SAHA for 1 hour and simultaneously subjected to heat shock at 43°C for 1 hour. Three replicates for each sample were generated and analyzed. RNA extraction, library preparation and sequencing were outsourced to Tsingke Biotechnology (Beijing, China). Reads counts were scaled to CPM (count per million). Then we used R package DESeq2 to identify the differentially expressed genes (DEGs). We set *p-*value less than 0.05 and log2 fold change greater than 0.585 or less than -0.585 as cutoff of significant DEGs. The original RNA sequencing (RNA-seq) data are uploaded in the open-access Gene Expression Omnibus (GSE228159, https://www.ncbi.nlm.nih.gov/geo/).

### Animal Studies

615 adult male mice were purchased from National Longitudinal Cohort of Hematological Diseases in China. MFC cells (1×10^6^) were injected subcutaneously into the back of 6- to 8-week-old male 615 mice. Tumors were measured by a caliper. When tumors reached a size of approximately 50 mm^3^, we randomly distributed the mice into six groups (six mice in each group): (1) untreated control, (2) hyperthermia alone, (3) SAHA (25 mg/kg bodyweight) alone, (4) LBH589 (10mg/kg bodyweight) alone, (5) SAHA (25 mg/kg bodyweight) + hyperthermia, and (6) LBH589 (10mg/kg bodyweight) + hyperthermia. And then, SAHA (25 mg/kg) was injected intra-peritoneally every day until day 21. LBH589 (10 mg/kg bodyweight) was injected intra-peritoneally every other day until day 21. Hyperthermia was given every other day until day 21. Tumor volume measured twice a week after the initial injection, and the volumes were calculated using the formula (length×width^2^/2). The mice were sacrificed on day 22 after tumor inoculation and the tumors were harvested. Experimental procedures involving animals were performed in accordance with guidelines of the Ethics Committee of Shanghai Jiao Tong University (A2022012).

### Immunohistochemistry

For histological examination, mouse tumors were fixed in 4% neutral buffered formalin phosphate (pH 7.0) and subsequently embedded in paraffin. Tumor paraffin sections were subjected to deparaffinization and rehydration using xylene and alcohol gradients. Antigen retrieval was performed in citrate buffer (pH 6.0) with a subsequent treatment of 3% H_2_O_2_ for 30 minutes to inhibit endogenous peroxidase activity. Following the pre-treatment steps, the sections were incubated overnight at 4°C with a cleaved caspase-3 antibody. Detection of the antibody was carried out using an Immunohistochemistry Kit (Sangon Biotech) in accordance with the manufacturer's instructions.

### Statistical Analysis

Statistical significances are reported in the Figures and in the Figure legends. Each experiment was performed three times independently. The values shown are the mean ± SEM, as indicated in each Figure caption. *P* values were determined using two-tailed unpaired t test or two-way ANOVA. N numbers are indicated in the Figure legends. Statistical analysis was performed using GraphPad Prism 8.0. *p* < 0.05 was considered statistically significant (*), *p* < 0.01 as highly significant (**), *p* < 0.001 as very highly significant (***), *p* < 0.0001 as extremely significant (****), and ns as not significant.

## Supplementary Material

Supplementary figures.

## Abbreviations

HSPs: heat stress proteins
H3K9ac: H3K9 acetylation
HDACs: histone deacetylases
HSP90: heat shock protein 90
DMEM: Dulbecco's modified Eagle's medium
FBS: Fetal Bovine Serum
SDS: sodium dodecyl sulfate
SDS-PAGE: SDS-polyacrylamide gel electrophoresis
CCK8: Cell Counting Kit-8
WB: Western Blotting
RT-PCR: real-time PCR
RNA-seq: RNA sequencing
CPM: count per million
DEGs: differentially expressed genes
HATs: histone acetyltransferases
siRNA: short interfering RNA
