# Supplementary material for: Inhibition of Heat Shock-Induced H3K9ac Reduction Sensitizes Cancer Cells to Hyperthermia
Source: Int J Biol Sci. 2023 Sep 11;19(15):4849–64. doi: 10.7150/ijbs.86384 (PMC10539700; doi:10.7150/ijbs.86384)
Supplement: Supplementary file 1 — Supplementary figures. [file ijbsv19p4849s1.pdf]

Supplementary data

Inhibition of Heat Shock-Induced H3K9ac Reduction Sensitizes Cancer Cells to Hyperthermia

Huiyun Lin[1], Yihui Song[1], Lingjun Song[2], Zilong Geng[1], Runtan Cheng[1], Yinrui Lei[1,], Fang Guo[1,*]*

Author information

Authors and Affiliations

Huiyun Lin, Yihui Song, Zilong Geng, Runtan Cheng, Yinrui Lei, Fang Guo

[1]Key Laboratory of Systems Biomedicine, Shanghai Center for Systems Biomedicine, Shanghai Jiao Tong University, 800 Dong chuan Road, Shanghai 200240, China.

Lingjun Song

[2]Pathology Center, Shanghai General Hospital, Shanghai Jiao Tong University School of Medicine, 100 Hai-Ning Road, Shanghai 200080, China.

*Corresponding authors

Correspondence to Fang Guo or Yinrui Lei.

Email: fguo@sjtu.edu.cn (Fang Guo) or to yrlei@sjtu.edu.cn (Yinrui Lei)

Tel: +86 18616213863 (Fang Guo) or to +86 13636550806 (Yinrui Lei)

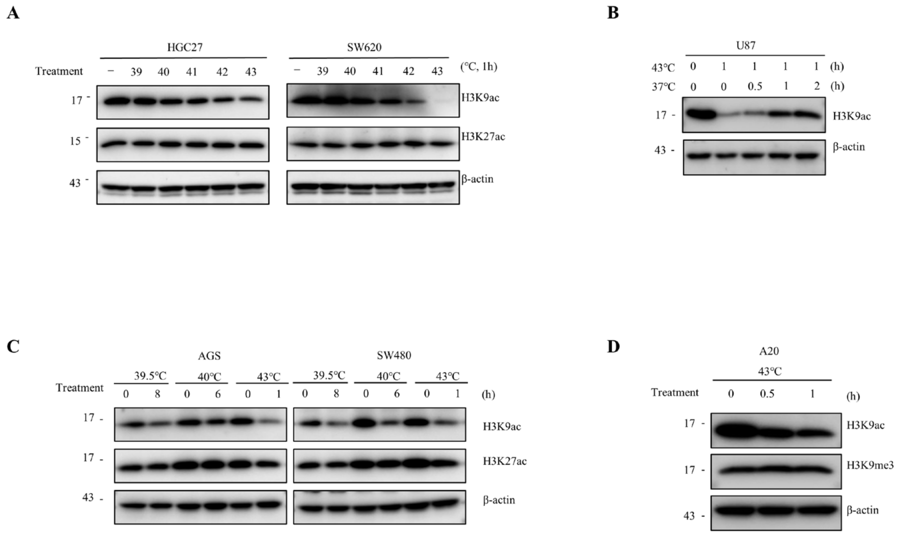

Figure S1. A HGC27 and SW620 cells were subjected to heat shock at the indicated temperature for 1 hour and analyzed by WB to evaluate the changes in acetylation at histone 3 lysine 9 and 27. **B** U87 cells were heat shocked at 43℃ for 1 hour and allowed to recover at 37℃ for the indicated periods, followed by WB to determine changes in acetylation at histone 3 lysine 9. **C** AGS and SW480 cells were exposed to heat shock at 39.5℃, 40℃ and 43℃ for the indicated times and analyzed by WB to assess the changes in acetylation at histone 3 lysine 9 and 27. **D** A20 cells were heat shocked at 43℃ for 30 min or 1 hour, followed by immunoblotting to determine alterations in acetylation and methylation at histone 3 lysine 9.

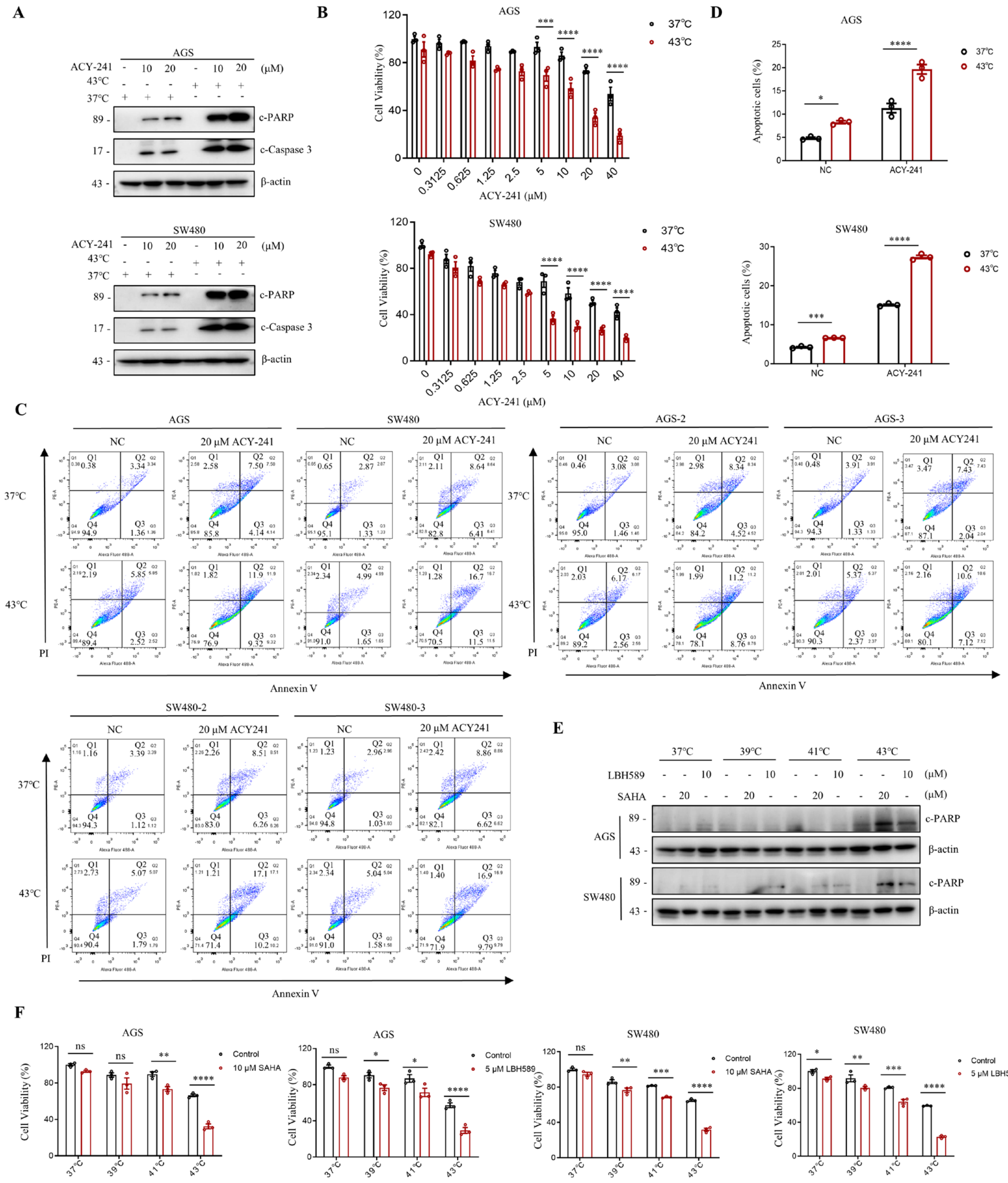

Figure S2. A-D AGS and SW480 cells were pre-treated with ACY-241 at the indicated concentration for 24 hours (A, C) or 48 hours (B), followed by heat shock at 43°C for 1 hour. Cells were fixed and stained for WB (A), subjected to the CCK8 assay (B), and analyzed by flow cytometry (C). Percentage of apoptotic cells was quantified by flow cytometry assay (D). N=3. Date: mean ± SEM. Statistical analysis: two-way ANOVA. *p-value <0.05, ***p-value <0.001, ****p-value <0.0001. All CCK8 experiments

reported only results with p-value < 0.001. **E-F** AGS and SW480 cells were pre-treated with SAHA and LBH589 at the indicated concentration for 24 hours (E) or 48 hours (F), followed by heat shock at 43°C for 1 hour, and cells were fixed and stained for WB (E) and CCK8 (F).

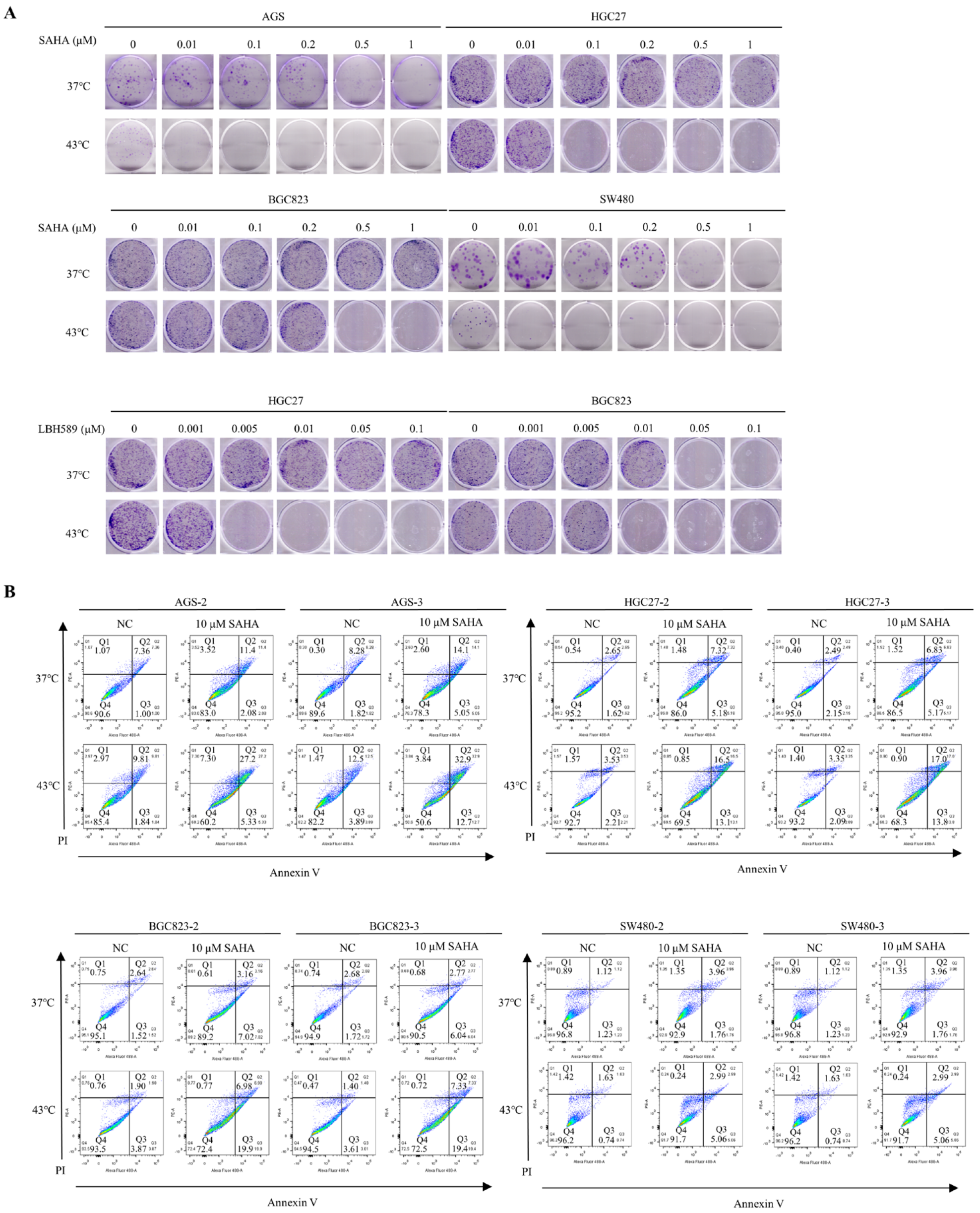

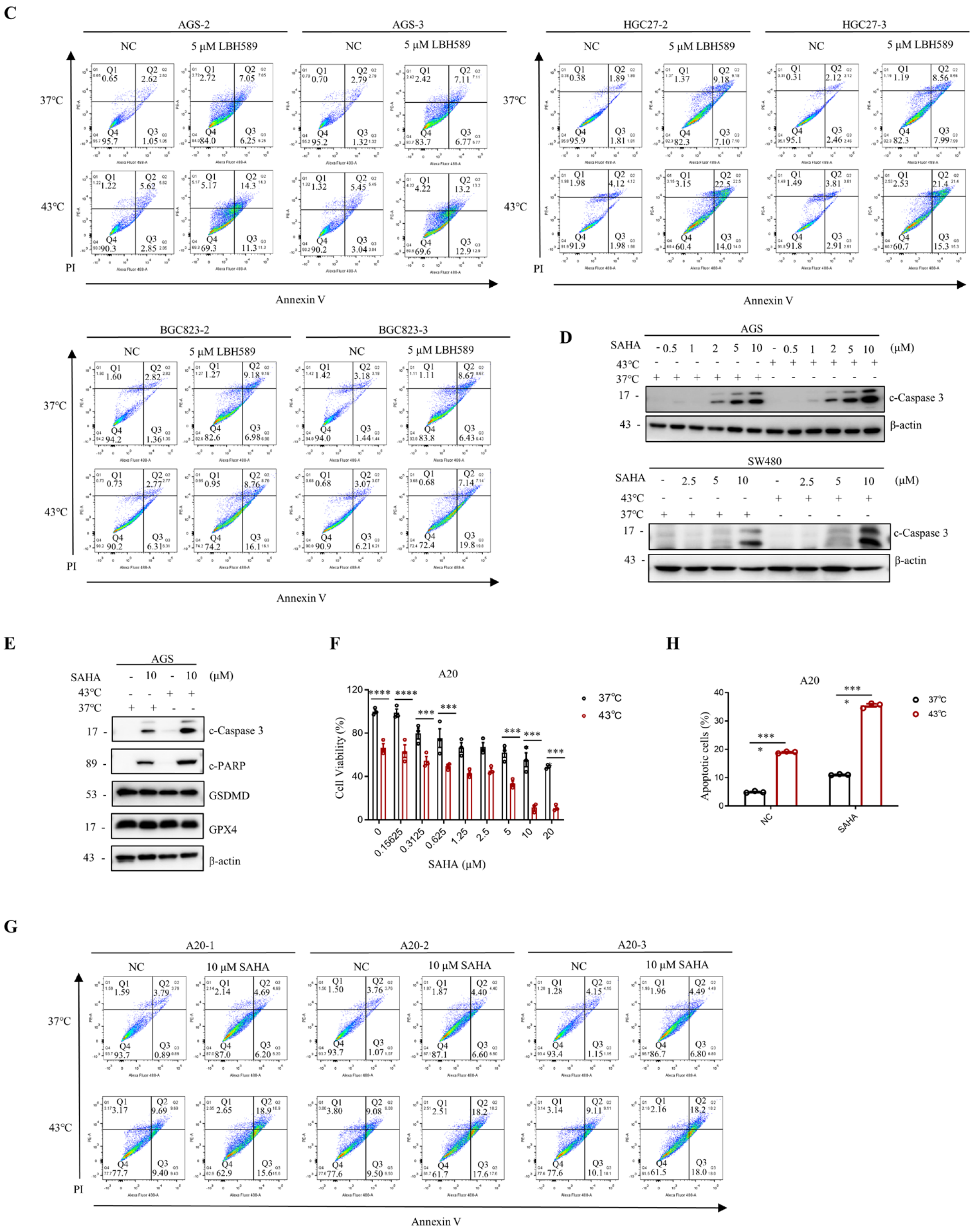

Figure S3. A Colony-formation assay. Cells were measured with crystal violet staining after treatment with SAHA or LBH589 at the indicated concentration combined with or without heat shock at 43°C. **B, C** Cells were pre-treated with SAHA (10 μM) (B) or LBH589 (5 μM) (C) for 24 h, then treated with or without heat shock at 43°C for 1 hour. The cells were fixed analyzed by flow cytometry. **D** AGS and SW480 cells were pre-treated with SAHA at the indicated concentration for 24 h, then treated with or without

heat shock at 43°C for 1 hour. WB was performed to assess changes in cleaved Caspase 3. **E** AGS cells were pre-treated with SAHA (10 μM) for 24 h, then treated with or without heat shock at 43°C for 1 hour. WB was performed to assess the changes in c-Caspase 3, c-PARP, GSDMD and GPX4. **F-H** A20 cells were pre-treated with SAHA at the indicated concentration for 24 h (G) or 48 h (F), then treated with or without heat shock at 43°C for 1 hour. The cells were fixed and subjected to the CCK8 assay (F), and analyzed by flow cytometry (g). Percentage of apoptotic cells was quantified by flow cytometry assay (H). N=3. Date: mean ± SEM. Statistical analysis: two-way ANOVA. ***p-value <0.001, ****p-value <0.0001.

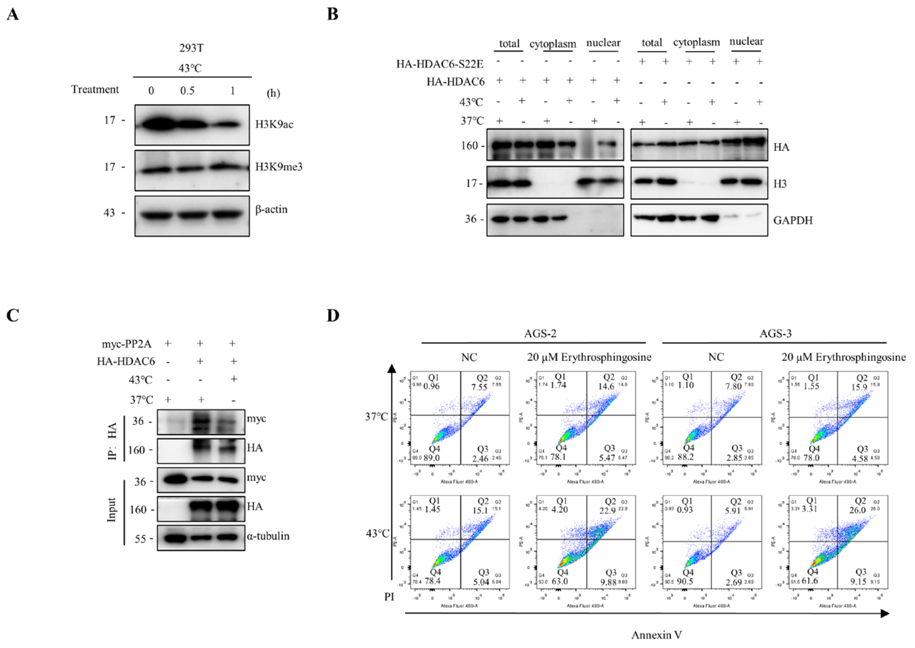

Figure S4. A 293T cells were heat shocked at 43°C for 30 min or 1 hour, followed by immunoblotting to determine changes in acetylation and methylation at histone 3 lysine 9. **B** HEK293T cells were co-transfected with HA-HDAC6-S22E and HA-HDAC6

plasmids, followed by heat shock for 1 h. Nuclear cytoplasmic fractionation assay was performed to separate nuclear and cytoplasmic fractions, which were analyzed by WB. **C** HEK293T cells were co-transfected with myc-PP2A and HA-HDAC6 plasmids and then subjected to heat shock at 43°C for 1 h. Co-precipitated proteins were detected by WB using anti-HA antibody after immunoprecipitation. **D** Flow cytometry analysis was performed on AGS cells treated with or without 1-hour heat shock at 43°C for 1 hour.

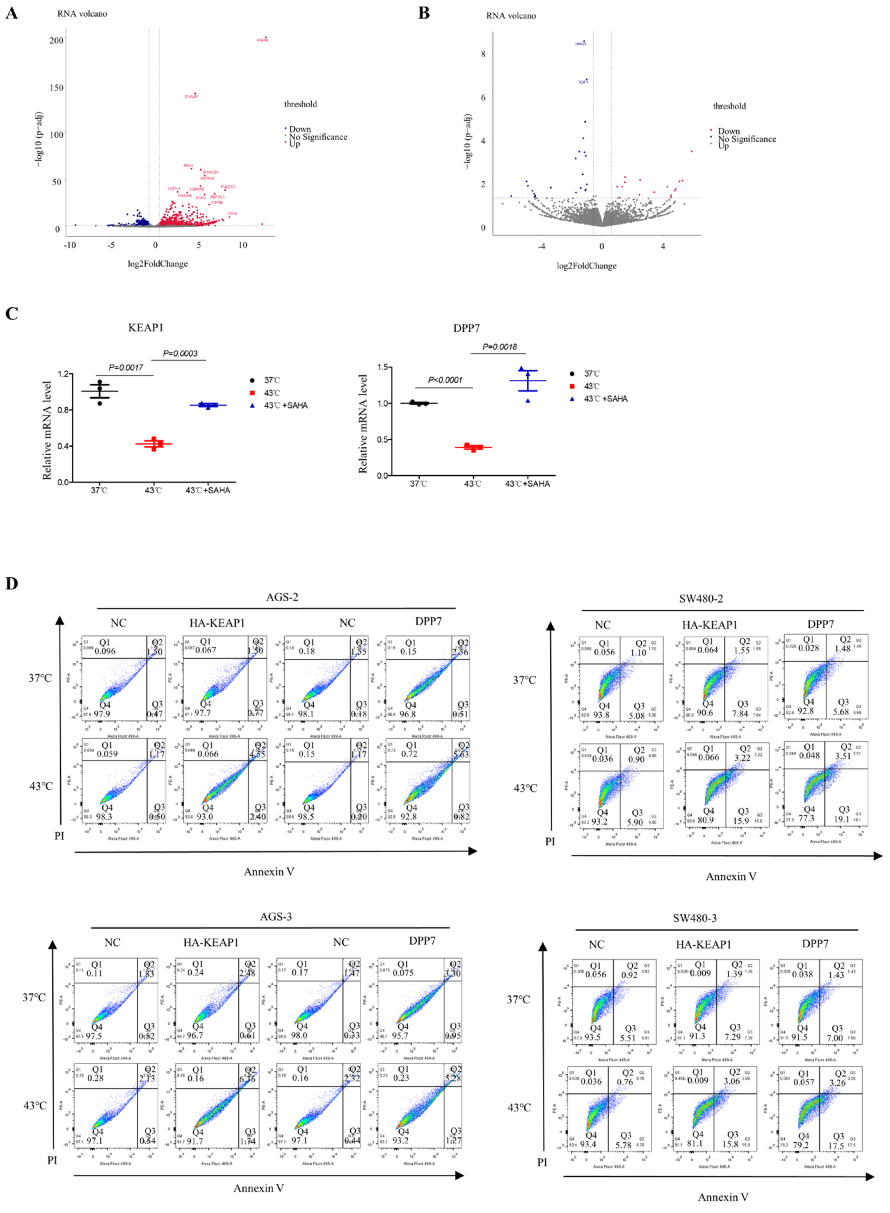

Figure S5. A, B RNA volcano plots show the genes regulated by heat shock. AGS cells were treated with or without heat shock at 43°C for 1 h (A) and in the presence of SAHA (0.02 μM) (B). N=3. **C** Real-time PCR was used to measure changes in mRNA expression levels of genes that were upregulated by heat shock at 43°C for 1 hour and subsequently downregulated by SAHA (0.02 μM). N=3. **D** Real-time PCR was used to measure changes in mRNA expression levels of KEAP1 and DPP7 in AGS cells treated with heat shock at 43°C for 1 hour in the presence of SAHA (0.02 μM). N=3. **E** Flow cytometry analysis was performed on AGS and SW480 cells transfected with HA-KEAP1 or HA-DPP7 plasmids, followed by treatment with or without heat shock at 43°C for 1 hour.

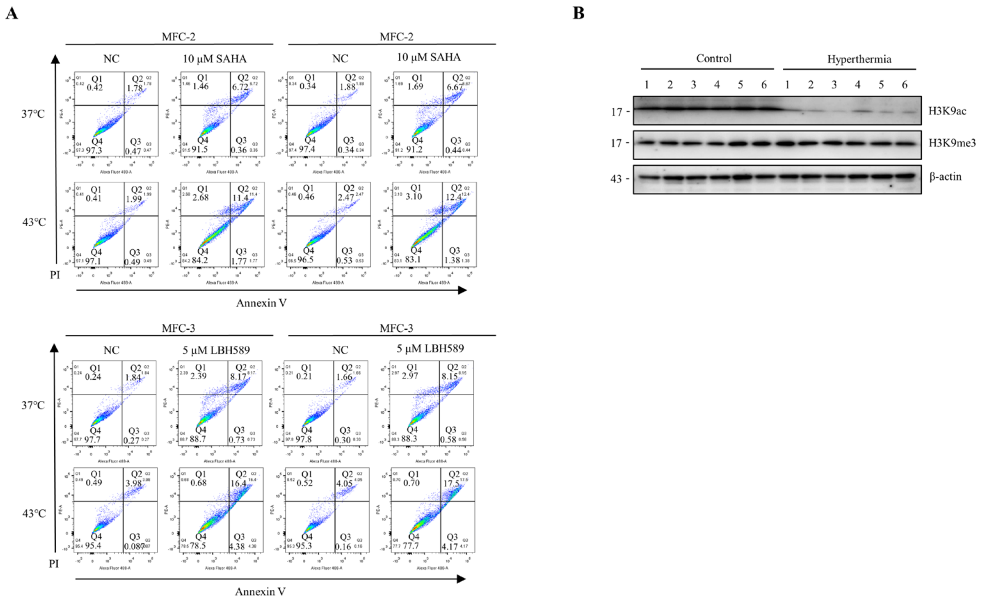

Figure S6. A MFC cells were pre-treated with SAHA (10 μM) or LBH589 (5 μM) for 24 h, then treated with or without heat shock at 43°C for 1 hour. The cells were fixed analyzed by flow cytometry. **B** Western blot showing H3K9ac changes under the

specific hyperthermia condition used in the mouse models. N=6.
